# Medical Opinion and Movement

**Published:** 1910-05-21

**Authors:** 


### Medical Opinion and Movement.

### Breast-feeding and Tuberculous Meningitis.

It is generally admitted that breast-feeding protects infants against infectious diseases, and in regard to tuberculosis a special relation is supposed to exist between the occurrence of the disease and feeding with cow's milk. According, however, to the statistics furnished by Dr. Zapport, of Vienna, tuberculous meningitis is far from rare among breast-fed infants. Of 125 infants who succumbed to the disease in the Hospital Sainte Anne at Vienna, 88 were breast-fed for at least three months, and 37 were fed artificially, representing a proportion of 70.4 per cent. to 29.6 per cent. In order to obtain a definite conclusion from these figures it is, of course, necessary to know approximately the proportion of breast-fed infants among the general population. From an inquiry made by Dr. Keller for this purpose from 1,300 mothers it was found that 398 had either not fed the infants naturally at all or only for the first few weeks, against 902 who had fed their infants for at least three months, representing a proportion of 36.6 per cent. to 69.4 per cent. In other words, the proportions are almost the same as for the children who had died from tuberculous meningitis.

### Spinal Anæsthesia and the Kidneys.

Contrary to the opinion of several enthusiasts for spinal anæsthesia, who have claimed for this method, among other advantages, that it does not induce albuminuria, Dr. Schwartz has found as a result of his observations on 60 patients submitted to spinal anæsthesia with stovaine that 47, or 78.33 per cent., showed renal changes more or less serious. In all these cases the urine was ascertained to be normal previous to operation. Similar results have also been reported since by others. Dr. Marcozzi has endeavoured by experiments on animals to ascertain the pathology of these renal changes. He finds that subcutaneous injections of non-toxic doses of stovaine are rapidly eliminated without any changes in the renal tissues or in the urine. Even with the subcutaneous injection of fatal doses of stovaine there is only a slight hyperæmia of the kidney, with a trace of albuminuria in the urine. On the other hand, when injected into the spine, even in non-toxic doses, the stovaine produces almost invariably marked changes in the urine (albuminuria, hyaline casts, and red corpuscles), and in the kidneys histological examination showed not only a more or less intense hyperæmia but also small hæmorrhages and lesions of the glomeruli and renal epithelium. From these facts the author concludes that the renal changes are not due to the direct action of the anæsthetic upon the kidneys but to some influence of the stovaine upon the nervous centres associated with these organs. He suggests that the stovaine introduced into the subarachnoid space forms an abnormal stimulus to these nerve centres, exciting the urinary secretion either by means of special secretory nerve fibres or through the blood-vessels.

### Diagnostic Signs for Organic Hemiplegia.

The difficulty frequently encountered in diagnosing organic hemiplegia from functional conditions which may closely simulate it has led neurologists to devote special attention to any distinguishing signs that may manifest themselves, and in consequence a number of new clinical phenomena of considerable diagnostic value have been discovered. In a contribution to *La Semaine Médicale*, Dr. J. Lhermitte gives an interesting *résumé* of the later additions to these differential signs. One of these consists in the "sign of the interossei," or "the sign of the fingers." To demonstrate this the patient is told to raise the paralysed arm. With the execution of this movement the fingers of the paralysed hand are involuntarily extended and stretched apart from one another like the opening of a fan, and assume again their original position when the arm is let fall. In some cases the phenomenon appears with passive elevation of the arm. This sign was discovered by Dr. Songues, and he found it present in 17 cases out of 29. The abduction and extension of the fingers result from contraction of the interossei muscles. The "thumb sign" of Klippel and Weil may be demonstrated in cases of organic hemiplegia with contraction. In a normal subject with the fingers half flexed and the muscles of the forearm relaxed, if they are slowly bent back into a position of extension the thumb also is involuntarily extended. In a case of hemiplegia with contraction, however, if the contracted fingers are passively extended the thumb is immediately flexed on the palm. Both of these signs only apply to cases of hemiplegia with contraction.

Dr. Raimiste has, however, drawn attention to two other signs for cases of flaccid hemiplegia, known as "the sign of the hand" and "the sign of associated adduction and abduction. The paralysed arm is laid flat on the bed and the forearm and hand are raised to the vertical, the hand being supported so as to be in the same plane with the forearm. While the attention of the patient is drawn away, the observer glides the supporting hand quietly down the forearm, and immediately the paralysed hand, deprived of support, falls abruptly, forming an angle of 130° to 140° with the forearm. This sign is immediately apparent after the stroke and while the patient is still comatose, whereas Dr. Raïmiste finds that in the cadaver soon after death, or in a subject deeply under chloroform, the hand maintains its vertical position. He supposes, therefore, that the fall of the hand is not due to a lack of muscular tone, but to an increase of tone in the flexor muscles of the hand. In hysterical hemiplegia too the sign is absent. The sign of associated adduction and abduction applies to the lower extremities, and is similar to the sign of complementary opposition first demonstrated by Hoover and already described in these columns. The patient is placed on a hard flat bed, so that there is no impediment to the movements of the legs. If now the legs are abducted and the patient is told to adduct the sound leg, and at the same time the movement is prevented by holding the leg firmly, the efforts of the patient are shown by an adduction of the paralysed leg, even though the patient is unable to execute any voluntary movement with it. Similarly with legs placed together, resisted abduction of the sound leg is accompanied by abduction of the paralysed leg. Another sign for the paralysed lower limb has been demonstrated quite recently by Dr. Néri, and like the "hand sign" of Dr. Raïmiste depends upon the hypertonicity of the flexor muscles. If the patient is placed on the back the sound limb can be elevated to an angle of 70° to 75° to the bed, while the paralysed leg can only be raised to an angle of 40° to 50°.

### New Trephining Instruments.

An American surgeon, Dr. W. H. Hudson, describes in *Surgery, Gynæcology, and Obstetrics* a new instrument—or rather a new series of instruments—for opening the skull. These consist of spiral burrs driven by a hand-brace, and the special advantage claimed for them is that they cease to cut, and indeed the brace ceases to drive, as soon as the opposing layer of bone becomes so thin as to be no longer rigid. As long as there is a firm resistant surface to cut against, they go on cutting; but, owing to their construction, it is alleged that injury to the brain, or even to the dura mater, is quite impossible. The set consists of a perforator, a dull pointed burr or follower, and an enlarging burr with a blunt centre. In addition to these there is added to the outfit a new forceps used to connect adjacent trephine holes. This is not constructed on the punch principle such as is exemplified in Hoffman's forceps and is generally in favour in Britain, but is of the nature of a shearing tool. It is claimed by the inventor of these instruments that not only is the operation of trephining rendered much less dangerous, but that it is also simplified and made much easier in execution.

### Terminal Arterial Anæsthesia.

Under this name Dr. Ransohoff describes in the *Annals of Surgery* a method of local anæsthesia which, though applicable only to a certain group of cases in limited areas of the body, is said to be perfect of its kind. The anæsthesia is induced by the injection of cocaine solution directly into the artery supplying the area to be anæsthetised. The resulting anæsthesia supervenes within two minutes, and is so absolute that amputation can be performed without the patient's knowledge, provided that the operation field is not within his range of vision. The following is the technique advised for man; it has been evolved by animal experimentation, and is said to be free from risk. The main artery supplying the part is exposed under infiltration anæsthesia. An Esmarch strap is now bound about the limb some distance above the point of proposed injection into the artery. The Esmarch should be used as in the Bier hyperæmic treatment; that is, to constrict the veins, but not tight enough to interfere with the arterial circulation. From 4 to 8 c.c. of a 0.5 per cent. cocaine in normal salt solution should be injected in the direction of the blood stream. The needle should be used as fine as possible. At the end of the operation the Esmarch is removed and the wound closed. Apparently no ligature is placed round the wounded artery. The method is held to be particularly applicable to the upper extremity, where the brachial, radial, or ulnar arteries may be exposed with little difficulty. The advantage of the process is said to be its absolute safety, due to diffusion of the cocaine solution into the tissues. The quantity of the drug used is so small that toxic effects are almost impossible.

### Results of Removing both Ovaries and Tubes.

The second part of Dr. A. E. Giles's extremely important paper on this subject, now published in the *Journal of Obstetrics and Gynæcology of the British Empire*, deals with points perhaps even more important than those considered in his first paper, noted in these columns already (The Hospital, April 9, 1910, p. 34). For the full discussion of his statistics, which is an admirable example of fair and scientific analysis, the original must be consulted; but his conclusions, which seem to be entirely warranted by the facts upon which they are based, are as follows: The removal of both ovaries and tubes has no marked detrimental effect upon the subsequent health, for 78 per cent. of the patients were in very good health afterwards, and a further 13 per cent., though suffering in different ways, were better than before the operation, making in all 91 per cent. who were quite well or at least improved. The condition of the general health is even better than it is after unilateral salpingo-oophorectomy. The likelihood of later trouble developing in connection with the uterus when that organ is left is relatively small, as such an occurrence took place in only seven cases out of 105. It is, therefore, worth while leaving the uterus in all cases where it appears to be healthy; for the removal of the uterus appears to increase the risk of the operation in inflammatory cases.

### The Artificial Menopause.

Menstruation continues after these operations in about 40 per cent. of the cases, the proportion being largest when the operation has been done for inflammatory disease. When menstruation persists after ovariotomy for tumours it is mostly in parovarian or intraligamentary cases. The inference is that some portion of ovarian tissue has remained behind in these cases. The characteristics of the artificial menopause produced by complete removal of both ovaries are as follows: Flushes of heat come on within three months in 80 per cent. of cases, and may go on for many years; probably the average duration is three or four years. The majority of patients (72 per cent.) retain their bodily vigour and energy. Those who complain of lack of energy and of being easily tired may be not necessarily all thus affected in consequence of the menopause. In only 10 per cent. of cases is there any mental depression evoked by the artificial menopause. In

68 per cent. the sex-instincts are unaffected; in 16 per cent. they are diminished; and in 16 per cent. they are lost after the operation. The artificial, like the natural, menopause is followed by some atrophy of the uterus and vagina, but not in all cases; 62 per cent. showed some change within two years, 73 per cent. within five years, and 82 per cent. after more than five years. The tendency to obesity is not so marked as after the natural menopause. There is no foundation for the view that the removal of the ovaries leads to masculine characteristics, such as growth of hair on the face, atrophy of the breasts, and deepening of the voice, except perhaps when the operation is done before the age of puberty.

### Epidemic Pemphigus Neonatorum.

Pemphigus neonatorum is still classed by Sir Malcolm Morris with the neurotic skin affections; but, syphilitic pemphigus altogether apart, he recognises that several causes may underlie this peculiar disease, particularly septic infections. In the *Bulletin of the Lying-in Hospital of New York* Dr. Schwartze describes an epidemic of 27 cases which occurred in that institution in four months. Of these babies nine died, but it is considered that three of these possibly did not die as a direct consequence of the pemphigus. In no case were the eyes or mouth affected, nor did the umbilicus ever appear to be the point of entry of the infection. Cultures made from the blebs before and after death showed only a staphylococcus; a similar observation has been made in this country by Maguire, though Adamson believes that a streptococcus is the real infecting organism. As regards ætiology, the most important finding seems to be in regard to the attending accoucheur. The first two cases were attended by two different physicians, and appear to have arisen spontaneously; but the next nine cases were all attended by one man. The contagious nature of the epidemic is further attested by the fact that shortly after the first cases were admitted the disease began to develop among children born in the hospital. Only when strict isolation was instituted did the disease become stamped out. The puerperium of all the mothers was normal, and the cases can, therefore, not be supposed to have started from maternal infection.

### Sclerosis of Uterus and Adnexa.

According to Brignolles, in *La Gyn.*, sclerosis of the uterus and adnexa, the ovaries in particular, is met with quite frequently apart from the menopause. Repeated attacks of pelvic congestion and progressive disturbance of the circulation occurring during the period of sexual life, and disabling the patient by the constant pain produced, eventually lead to the establishment of changes in the genital apparatus which terminate in sclerosis. The author estimates that 16 per cent. of gynæcological troubles are due to ovarian sclerosis. The disease is of varying degree, as is the suffering, but profound nervous and general systematic disturbances are met with even in the young girl. Pain is a most important symptom, which varies in degree and occurs usually at the period. It may, however, occur ten days before menstruation, and in chronic cases extend over most of the monthly cycle. Nausea and vomiting may accompany the pain. Local symptoms vary and include dysmenorrhœa, with intractable hæmorrhages following the period, metrorrhagia, amenorrhœa, and leucorrhœa. The ovaries may be found prolapsed either laterally or posteriorly, and may be either enlarged and cystic or smaller than normal and sclerosed. The uterus is usually enlarged, indurated, often retroflexed, but generally mobile. General symptoms include constipation, membranous diarrhœa, indigestion of various kinds, intercostal and spinal neuralgia, and pain on defæcation and urination during the congestive period. Exertion causes severe lumbar pain. Nervous symptoms include excitement and neurasthenic phenomena, irritability, and bad temper, especially in the thin subject, whereas the stout subject is usually listless and lies languid in fear of increased suffering. In both types there are often changes in character and intelligence. The author thinks that most cases of sclerosis are not due to gonorrhœa or other infection.

### Abdominal Hyperæsthesia.

Loeper and Esmonet maintain in *La Presse Médicale* that the para- and sub-umbilical regions of the abdomen are the true areas of sensibility for the intestinal mass. Moderate pressure should elicit no pain at these points in a normal individual, but in persons suffering from various intoxications, dyspepsia, and enteritis, pain will be evoked which may amount to agony, and is often accompanied by irradiations into remote parts, aortic palpitations, modifications of arterial tension, attacks of pseudo-angina, purpura, vomiting, spasms, and tinnitus. This deep abdominal hyperæsthesia may be attributed to one of two principal causes, intestinal or general. It is often met with in abdominal maladies apart from all inflammatory phenomena, and would seem to arise from purely mechanical disturbances. It occurs, therefore, frequently in visceroptosis, the abdominal mass exerting a constant pull on the sympathetic filaments, and it is seen in certain glandular and spinal affections where the nerve plexuses are involved. In most cases, however, the hyperæsthesia is of purely inflammatory origin, digestive disturbances, fermentation, dyspepsia, intestinal stasis, atony consecutive to infectious maladies or general malnutrition, true enteritis, and infected cancer, all being causes of irritation to the sympathetic centres. In other cases the reaction of the nervous system would seem to be due to a series of more general causes. Thus tabes, nervous affections, auto-intoxication, and general infections may play their part. Or, again, abdominal hyperæsthesia may occur apart from any characteristic disease, in which case it is a true neurosis, though the possibility of the existence of some latent hereditary taint or auto-intoxication must not be overlooked. On the other hand, hypæsthesia and anæsthesia are met with in diabetics and tabetics, and should be sought for.

